# Review of *Computational Systems Biology of Cancer*

**DOI:** 10.1186/1475-925X-12-76

**Published:** 2013-08-06

**Authors:** Eric Bullinger, Monica Schliemann

**Affiliations:** 1 Department of Electrical Engineering and Computer Science (Montefiore Institute) and GIGA (Interdisciplinary Cluster for Applied Geno-proteomics), Université de Liège, Liege, Belgium

## Book details

*Computational Systems Biology of Cancer* by Emmanuel Barillot, Laurence Calzone, Philippe Hupé, Jean-Philippe Vert and Andrei Zinovyev. Chapman & Hall/CRC. Series: Mathematical & Computational Biology. Published: 25 August 2012–461 Pages. Accompanying website: http://www.cancer-systems-biology.net/.

## Introduction

The book presents an overview of systems biology applied to cancers, from the experimental part over bioinformatics aspects up to dynamical modelling. Thus, it covers a large variety of foundations and methods, which are necessary for the understanding of cancer from a computational systems biology angle as cancers are complex and robust dynamical systems.

## Summary of content

The book is divided into twelve chapters plus appendices. The book also contains lists of acronyms, genes, software and databases, as well as a glossary and a very extensive bibliography. The introduction gives a nice overview cancer, system biology and why systems biology approaches are necessary for medicine in the treatment of cancer.

Chapter 2 and Appendix 1 introduce readers, with limited biological knowledge, to molecular biology from gene expression to epigenetics and signal transduction. Some parts of the appendix are written in the style of an extensive glossary. A specificity of cancer biology is the importance of mutations. These are described in detail, both at the genetic level as well as at the protein level, i.e. how modified proteins affect cellular regulation underlying the systems aspect of cancer.

Chapter 3 describes nicely and in an easily understandable manner high-throughput experimental techniques for the quantification of DNA, RNA, proteins and their interactions. Additionally, computational quantification methods are also presented. Some of these would be more easily understandable if a short motivation was added, e.g. for the denominator of BAF.

Chapter 4 gives an overview of bioinformatics tools and standards and seems to be written for a more theoretical audience. This applies to Section 4.1 in particular, due to its mathematical notation.

Chapter 5 discusses how features can be extracted from large-scale data, e.g. genome-wide mRNA or protein amount. Different methods are presented and well illustrated. These analyses are a key step between the high-throughput experiments and their medical interpretation. The mathematical foundation of the latter is thoroughly covered in Chapter 6. Methods of statistical inference for feature detection, for example, are presented.

Chapter 7 gives a good overview of dynamical mathematical modelling approaches. The role of positive and negative feedback loops are very clearly presented in the example of small cell cycle models. Chapter 8 gives a very detailed overview of published dynamical models involved in cancers, from growth and death signalling to energy metabolism.

Robustness is the topic of Chapters 9 and 10, in the first chapter from a biological point of view, in the second one from a mathematical one. Chapter 11 presents approaches targeting the fragility of networks and how these can be used for predicting drug targets.

## Analysis and evaluation of the book

The book gives a very good overview of the broad and complex field of systems biology approaches dealing with cancer. The authors give a very comprehensive introduction to this field, particularly for graduate students and researchers with a background in mathematics, computer science or engineering.

Chapters dealing with high-throughput experiments use real data while the mathematical modelling chapters do not contain experimental data. Therefore the process from a real biological problem, with experimental data, to a fully developed mathematical model is not described in this book.

Reading the book is free flowing and motivates one to look into cited references in order to dig deeper. The book also has aspects of a reference book, as can be seeing from the glossary and several text boxes with definitions. This aspect could be strengthened significantly if all definitions could be easily found either in the glossary or via the index. For example Box 4.3 defines, among others, metabolic and signalling pathways. The first term is not in the glossary, while the second is there with a very similar text to what is in the chapter. Also, not all genes and glossary items are listed in the index. With a few questions after each chapter and an accompanying website that includes all the figures of the book as well as several scripts and datasets, the book can also be used for teaching systems biology to an engineering audience.

Readers could find it helpful if there was a better organization of the different glossary-style parts as well as more cross-references, for example from the glossary back to the individual book sections. This book could then be used as a hand book for looking up definitions and short descriptions of the different topics of the systems biology of cancer.

## Conclusion

The book is an excellent starting point for readers with a theoretical background interested in cancer systems biology. The book presents an extensive list of methods as well as a large number of definitions in the glossary or in text boxes. The methods are well illustrated, with a good choice of biological examples. The often condensed presentation is enough to give an insight, but not to gain a thorough understanding. Here for, the extensive list of references is an excellent starting point.

## Competing interests

The authors declare that they have no competing interests.

## Authors’ contributions

Both authors contributed equally.

